# In Situ Determination of Chlorella Concentration Using Single Entity Electrochemistry

**DOI:** 10.3390/s26030915

**Published:** 2026-01-30

**Authors:** Changhui Lee, Gayeon Lee, Jun Hui Park

**Affiliations:** Department of Chemistry, Chungbuk National University, Cheongju 28644, Republic of Korea

**Keywords:** *Chlorella*, collision, single entity electrochemistry, ultramicroelectrode, migration

## Abstract

Harmful algal blooms pose significant risks to water resource management and aquatic ecosystem health, rendering early detection of algal bloom proliferation essential. In this study, we present an electrochemical strategy for the real-time detection of individual *Chlorella* cells using the single-particle collision method at an ultramicroelectrode (UME). The detection principle relies on monitoring changes in the redox probe flux at the UME induced by attachment of the target. Both diffusional and migrational transport were considered to promote particle collision at the UME. Detection sensitivity for negatively charged microalgae was enhanced by exploiting migration effects. To control migration strength, neutral and charged redox probes were selected, and the ionic strength was adjusted to tune electrostatic attraction, yielding microalgae capture on the UME with a collision frequency that depended on the solution composition. Conversely, migration was suppressed by increasing the ionic strength, and inverse migration was implemented, and resulting collision responses were compared. Furthermore, COMSOL Multiphysics simulations were used to estimate the size of detected *Chlorella* cells. The collision frequencies expected from diffusion and migration were compared with the experimental values, and a calibration curve relating collision frequency to *Chlorella* concentration was established. Consequently, this methodology provides a promising platform for the early monitoring of algal blooms by simultaneously determining microalgal size and concentration.

## 1. Introduction

Harmful algal blooms (HABs) in lakes and rivers worldwide present a critical challenge to water resource management and security [1,2,3]. Toxins such as microcystin produced by cyanobacteria not only degrade water quality but also pose serious threats to aquatic life, livestock, and human health upon release into the water [4,5]. Consequently, detection of harmful algae and their toxins is a priority for environmental management and public safety. Furthermore, monitoring the progression of algal blooms is critical because massive proliferation can rapidly deplete dissolved oxygen through respiration during non-photosynthetic periods, thereby exerting adverse effects on aquatic ecosystems [6,7]. Therefore, this study focuses on the electrochemical detection of the unicellular microalga *Chlorella* as a representative model. Additionally, *Chlorella* serves as a surrogate for microcystin-producing cyanobacteria because of shared physical and electrical characteristics. For instance, *Microcystis aeruginosa*, a primary microcystin-producing cyanobacterium, typically ranges from 2 to 8 µm in size, while *Chlorella* measures approximately 2 to 10 µm [8,9]. Both species exhibit elliptical or spherical morphologies and possess negative zeta potentials [10,11]. These shared properties justify using *Chlorella* as a surrogate for microcystin-generating organisms.

Conventional methods for microalgae detection include microscopic counting, chlorophyll absorbance measurement by UV-Vis spectrophotometry, and polymerase chain reaction (PCR) [12,13,14,15,16,17]. While UV-Vis spectrophotometry is widely employed for bulk chlorophyll quantification, its accuracy is often compromised in complex environmental samples [18,19]. For instance, chlorophyll-a exhibits strong light absorption near 420 nm, which significantly overlaps with the absorbance spectrum of chromophoric dissolved organic matter (CDOM), originating from decaying plants or algae [20,21]. Consequently, the less sensitive absorption peak at 680 nm is preferred to enhance selectivity. Nonetheless, nanoscale contaminants such as nanoplastics or metal oxide particles induce light scattering or absorb light in the Vis-NIR region (e.g., 750 nm), thereby interfering with accurate absorbance measurements and baseline correction [22,23,24]. Recent microfluidic technologies offer precise control and high detection sensitivity [25,26]. However, these approaches require complex chip fabrication and sophisticated fluid-handling equipment, which impose inherent limitations in terms of system complexity and cost. Moreover, in flow-cell-based methods, the signal can depend on the cell trajectory within the channel, so cells of the same size may produce different responses depending on their lateral position [27]. In addition, achieving high sensitivity generally requires an aperture size comparable to the target size, and small apertures are prone to clogging by impurities [28,29].

To address these challenges, electrochemical detection via the single-particle collision method using an ultramicroelectrode (UME) was conducted. UMEs are defined as electrodes with at least one dimension (e.g., radius) of 25 µm or less, for which the steady-state mass transfer is readily established. The small geometric area of a UME minimizes capacitive charging current, providing high signal to noise in cyclic voltammetry even at relatively high scan rates. In chronoamperometry, UMEs rapidly attain a constant faradaic current because mass transport is dominated by radial diffusion. This steady state behavior is described by the Shoup–Szabo expression [30,31,32,33,34]. In contrast, for electrodes larger than approximately 100 µm, planar diffusion becomes dominant and the current decay is described by the Cottrell equation. Thus, using a UME, current changes arising from collisions of individual particles with the electrode can be readily detected [35,36,37,38,39,40]. This collision-based analytical approach is commonly referred to as Single-Entity Electrochemistry (SEE) and is widely utilized to determine particle concentration, size distribution, and intrinsic electrochemical activity [41,42,43,44]. Notably, the capability to detect single entities in real-time without complex pretreatment is well suited to complement limitations of existing *Chlorella* detection methods. Moreover, this method enables detection by simply immersing a UME in the sample solution, without requiring complex device fabrication. This simple configuration offers advantages in instrument miniaturization, cost-effectiveness, and potential applicability to field-deployable system.

Consequently, the detection of *Chlorella* was achieved in this study by applying the single-particle collision method to analyze the abrupt current fluctuations arising from particle–electrode collisions. Furthermore, specific electrolyte conditions that induce migration were employed to enhance the collision frequency, thereby improving detection sensitivity. The size of colliding *Chlorella* was estimated via COMSOL Multiphysics simulations by matching the measured current changes. The collision frequency as a function of cell concentration was also investigated. This methodology enables real-time detection of *Chlorella* in aqueous environments without the need for pretreatment. Ultimately, it may serve as a monitoring platform for early assessment of algal bloom progression by estimating both the size and concentration of the algal cells.

## 2. Materials and Methods

### 2.1. Reagents and Materials

All aqueous solutions were prepared using Millipore water (18.2 MΩ∙cm). Ferrocenemethanol (FcMeOH, 97%) and potassium ferrocyanide trihydrate (K_4_[Fe(CN)_6_]·3H_2_O, ≥99+ %) were purchased from Thermo Fisher Scientific (Waltham, MA, USA). Potassium hexacyanoferrate (K_3_[Fe(CN)_6_], 98+ %) and potassium nitrate (KNO_3_, 99%) were obtained from Alfa Aesar (Ward Hill, MA, USA). *Chlorella vulgaris* was obtained from the National Institute of Biological Resources (Incheon, Republic of Korea). Borosilicate glass capillaries (1.5 mm outer diameter × 0.75 mm inner diameter) were purchased from Sutter Instrument (Novato, CA, USA). Pt wires (diameter: 10 µm and 25 µm) were purchased from Goodfellow (Devon, PA, USA).

### 2.2. Instruments

Electrochemical experiments were conducted using a CHI 400A potentiostat (CHI Instruments, Austin, TX, USA) utilizing three-electrode systems. All electrochemical experiments were conducted inside a Faraday cage to minimize noise caused by external interference. The electrode assembly consisted of a Pt ultramicroelectrode (UME, diameter: 10 µm or 25 µm) as the working electrode, accompanied by an Ag/AgCl electrode (in saturated KCl) reference electrode and a Pt wire (diameter: 1 mm) counter electrode. Morphological characterization was performed using a SNE-4500M (SEC Co., Ltd, Suwon, Republic of Korea) scanning electron microscope (SEM) at acceleration voltage of 20 kV. *Chlorella* cells were collected and washed using a CF10 Centrifuge (DAIHAN Scientific Co., Ltd., Wonju, Republic of Korea), and their concentration was quantified using a Cary 8454 UV-Vis spectrophotometer (Agilent Technologies Inc., Santa Clara, CA, USA).

### 2.3. Preparation of the Pt-UME

The UME was prepared according to an established laboratory protocol adapted from the method described by Wightman [45]. Briefly, Pt wire (diameter: 10 µm or 25 µm) was sealed in a borosilicate glass capillary washed with hexane, toluene, IPA, ethanol, and water. The electrode was polished using silicon carbide abrasive sandpaper (400, 1200, 2000 and 5000 grit; R&B Co. Ltd., Daejeon, Republic of Korea) until a mirror-like surface was observed. Before each electrochemical experiment, all UMEs were polished using 5000 grit silicon carbide (SiC) sandpaper.

### 2.4. Parameters of Finite Element Simulation

Finite element simulations were performed using COMSOL Multiphysics 6.1 (COMSOL Inc., Burlington, MA, USA) with the Electroanalysis module. The computational work was carried out on a workstation equipped with an Intel Core i9-13900K CPU, NVIDIA GeForce RTX 3070 GPU, and 64 GB of RAM, operating under Windows 11. A 2D-axisymmetric geometry was employed to model the electrochemical cell. The simulation domain consisted of a 50 µm × 50 µm square region representing the bulk electrolyte, with the UME defined as a boundary of 5 µm radius located on the bottom boundary at the axis of symmetry (r=0). To model physical blocking by a single cell collision, a semicircular domain representing *Chlorella* was placed on the symmetry axis in direct contact with the electrode surface. The bulk concentration of the redox mediator was set to 1 mM, and the diffusion coefficient was set to 7.6 × 10^−10^ m^2^s^−1^. Mass transport was modeled by the Nernst–Planck equation, and electrode kinetics were described using the Butler–Volmer equation.

## 3. Results and Discussion

Single particle detection at UMEs is based on monitoring transient current changes arising from particle–electrode collisions. When a constant potential is applied to sufficiently drive a redox reaction (e.g., oxidation or reduction) at the electrode surface, a steady-state current is established under mass transfer limitation as redox species are transported to the electrode surface by radial diffusion. Under these conditions, the collision of a non-conductive particle hinders the diffusion of the redox probe by physically blocking part of the electroactive area and introducing steric obstruction, resulting in an abrupt decrease in the absolute value of the current. As the collided particle remains attached at the electrode surface, the current re-establishes to a new steady-state corresponding to the reduced mass transfer of the redox probe. This transition arises from hindered diffusion of redox species due to steric obstruction and a lower consumption rate of the redox probe, manifesting as a distinct staircase-like step response for each collision event. This electrochemical blocking principle enables the discrete detection of individual collision events and was employed in this study to detect *Chlorella* suspended in aqueous media.

**Figure 1:**
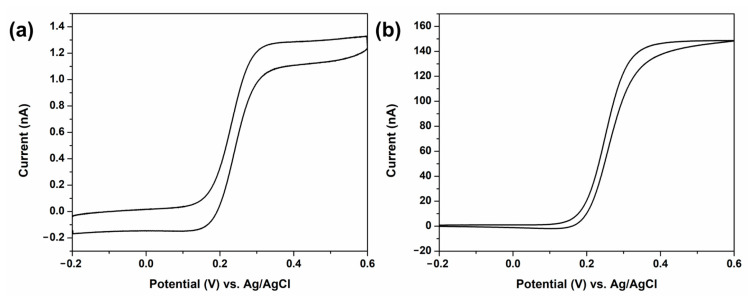
Cyclic voltammograms in aqueous solution containing (**a**) 1 mM FcMeOH and 0.5 mM KNO_3_ and (**b**) 100 mM K_4_[Fe(CN)_6_] using a 10 µm Pt-UME. The experiments are conducted at a scan rate of 50 mV/s.

Although *Chlorella* is a living biological entity with non-motile properties due to the absence of flagella, its physicochemical properties relevant to this method are comparable to those of other algae of similar size. Specifically, *Chlorella* cells predominantly exist as discrete unicellular particles with a near-spherical morphology, except under highly concentrated conditions. In addition, like most microalgae, *Chlorella* cells possess a predominantly negative surface charge at near-neutral pH due to the ionization of carboxylate and phosphate groups on the cell wall. Thus, this negative charge facilitates migration of the cells toward the electrode, thereby increasing the collision frequency. To induce migration, the region near the electrode surface should be locally positively charged via a redox reaction that generates positively charged species or consumes negatively charged ones. For migration-based detection system, FcMeOH and ferrocyanide solutions were employed as redox probes to evaluate cells collision tendencies of *Chlorella* cells as a function of electrolyte composition and ionic charge of the redox species. FcMeOH, a neutral species under near-neutral pH conditions, is oxidized at the electrode surface to generate the cationic ferrocenium species. Similarly, anionic ferrocyanide ([Fe(CN)_6_]^4−^) is oxidized to ferricyanide ([Fe(CN)_6_]^3−^), which is less negatively charged. Figure 1 presents the cyclic voltammograms (CVs) for both redox species. As shown in Figure 1a, the oxidation of FcMeOH initiates above 0.2 V, reaching a steady-state limiting current at potential ≥ 0.4 V. A comparable oxidation profile was observed for K_4_[Fe(CN)_6_] oxidation within a similar potential range (Figure 1b). Therefore, the *Chlorella* detection experiments were conducted at an applied potential of 0.5 V. This potential is sufficiently positive relative to the half-wave potential of FcMeOH oxidation to establish a steady-state current, which is required to resolve current decreases induced by *Chlorella* collision events. In addition, applying potentials more positive than 0.5 V leads to the onset of the oxygen evolution reaction (OER), introducing additional faradaic processes that can increase background noise and compromise collision-based detection. Accordingly, 0.5 V was selected to enable stable FcMeOH oxidation while avoiding side reactions.

**Figure 2:**
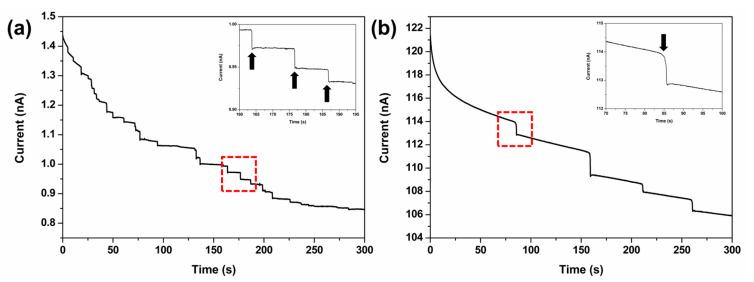
Amperometric *I*–*t* curves were obtained using a 10 µm Pt-UME in aqueous solution containing (**a**) 1 mM FcMeOH and 0.5 mM KNO_3_ with *Chlorella* and (**b**) 100 mM K_4_[Fe(CN)_6_] with *Chlorella* at +0.5 V (vs. Ag/AgCl). The inset images provide an enlarged view of the region outlined in the red box, with arrows indicating the step current generated by *Chlorella* collisions. The concentration of *Chlorella* used in both experiments is 9.08 fM.

To prepare the samples for electrochemical analysis, *Chlorella* cells were separated from the culture media via centrifugation, washed briefly with distilled water, and subsequently resuspended in the electrolyte solution. The chronoamperometric *I*–*t* response in Figure 2a, recorded in FcMeOH solution in the presence of *Chlorella,* exhibits discrete staircase-shaped current decreases corresponding to individual collision events. The clear staircase signals indicate that the collided *Chlorella* cells are well attached on the electrode surface, establishing a sustained blockage of mass transfer. Similarly, the chronoamperometric *I*–*t* response in Figure 2b, recorded in K_4_[Fe(CN)_6_] solution in the presence of *Chlorella,* exhibits staircase-shaped current decreases with a notably lower collision frequency than in the FcMeOH system. The discrepancy in collision frequency is attributed to differences in migration strength. The strength of migration reflects the electric field gradient developed in the vicinity of the electrode. At a constant current density, the electric field is inversely proportional to the solution’s conductivity, which scales directly with ionic strength. Consequently, maintaining a low ionic strength is critical for effectively inducing migration. In the FcMeOH solution, the ionic strength was maintained at 0.5 mM by adding 0.5 mM KNO_3_ as supporting electrolyte. In contrast, in the K_4_[Fe(CN)_6_] solution, dissociation and ion pairing yield multiple charged species, including [Fe(CN)_6_]^4−^, K[Fe(CN)_6_]^3−^, and K^+^, resulting in a significantly higher ionic strength. The enhanced ionic atmosphere at elevated ionic strengths hinders ion movement through relaxation and electrophoretic effects, thereby minimizing the contribution of electromigration. Furthermore, because *Chlorella* cells tend to sediment due to their density relative to the medium, a robust migrational force is required to counteract gravitational settling and effectively draw the cells toward the electrode surface.

The observed average step magnitudes for FcMeOH (14 pA) and [Fe(CN)_6_]^4−^ (1.07 nA) correspond to an approximately 76-fold difference. This experimental ratio is consistent with the theoretical prediction based on the 100-fold concentration difference and the 1.2-fold higher diffusion coefficient of FcMeOH, confirming the validity of our single-entity detection system. Furthermore, the slight discrepancy between the theoretical and experimental ratios may be attributed to enhanced mass transfer of FcMeOH by migration. In the low ionic strength regime (0.5 mM KNO_3_), migration is not negligible and increases the total probe flux above the diffusion limited value.

**Figure 3:**
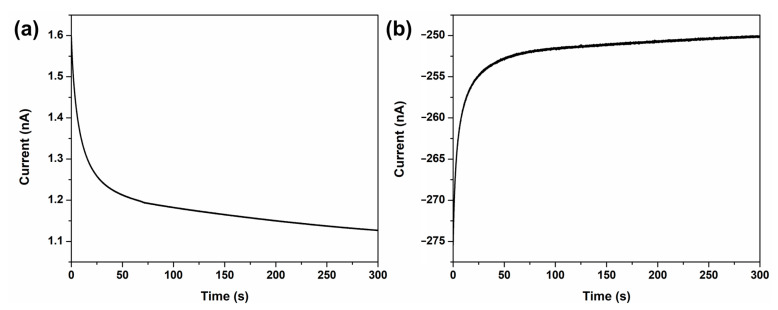
Amperometric *I*–*t* curves were obtained in aqueous solution containing (**a**) 1 mM FcMeOH and 50 mM KNO_3_ with *Chlorella* using a 10 µm Pt-UME at +0.5 V (vs. Ag/AgCl) and (**b**) 100 mM K_3_[Fe(CN)_6_] with *Chlorella* using a 25 µm Pt-UME at −0.2 V (vs. Ag/AgCl). The concentration of *Chlorella* used in both experiments is 9.08 fM.

To demonstrate that *Chlorella* detection at the UME is governed primarily by migration, additional experiments were conducted in which the KNO_3_ supporting electrolyte concentration was varied while the FcMeOH concentration was held constant. Additional measurements were also performed using an electrolyte containing ferricyanide as an alternative redox probe. Figure 3a shows the chronoamperometric *I*–*t* curve recorded in a solution containing 1 mM FcMeOH and 9.08 fM *Chlorella*, in which the KNO_3_ concentration was increased to 50 mM. However, no staircase-like current decreases were observed. This result indicates that increasing the KNO_3_ concentration raises the ionic strength and thereby strongly attenuates migration-driven collision*s*.

In addition to electrolyte composition, electrode dimensions also play an important role in the electrochemical detection of *Chlorella* cells. Ultramicroelectrodes with diameters of 10–30 µm can be used for *Chlorella* detection. However, the primary limitation arises from signal magnitude. As the electrode size increases, the relative current change induced by a single collision decreases, reducing detection sensitivity. Consequently, reliable detection requires that the ratio of electrode diameter to particle diameter be smaller than approximately 20. On this basis, for *Chlorella* cells with characteristic diameters of ~2 µm, electrodes with diameters below ~40 µm are expected to be suitable.

To theoretically interpret the results shown in Figure 3a in comparison with Figure 2a, the combined contributions of diffusion, migration, and convection to mass transport were evaluated. The total flux of the species in an electrochemical system is governed by the Nernst–Planck equation, expressed as follows:

(1) $$J=-D\nabla C-\frac{zFDC}{RT}\nabla \varphi +Cv$$

In this equation, J denotes the total flux of the species, D is the diffusion coefficient of the species, ∇C represents the concentration gradient of the species, and z corresponds to the charge of the ion. Additionally, F,R, and T represent the Faraday constant, the gas constant, and the absolute temperature, respectively, while φ indicates the electric potential and v denotes the fluid velocity. Because the solution was unstirred during the experiments, the convection term (Cv) was considered negligible. The diffusion contribution, described by Fick’s first law term (−D∇C), is expected to be comparable to that in Figure 2a because the same FcMeOH concentration was used. However, the migration term ($-\frac{zFDC}{RT}\nabla \varphi$), differs substantially because changing KNO_3_ supporting electrolyte concentration alters the potential gradient in solution. In electrochemical measurement, a supporting electrolyte is typically added to suppress the migration of charged species, so that mass transfer is governed primarily by diffusion. Accordingly, increasing the KNO_3_ concentration diminishes migration, and diffusion-driven *Chlorella* transport alone does not produce staircase current response, even though diffusion flux remains operative in the absence of migration. This is attributed to the fact that the cells are not effectively retained at the electrode surface. These results confirm that migration is essential for transporting negatively charged *Chlorella* to the electrode and that the resulting electric field retains the *Chlorella* cells at the electrode surface.

Similarly, Figure 3b shows the chronoamperometric *I*–*t* curve recorded in a solution containing 100 mM K_3_[Fe(CN)_6_] and 9.08 fM *Chlorella*. Reduction in ferricyanide in the vicinity of the electrode generates a locally negative electric field that electrostatically repels the negatively charged cells, thereby suppressing collisions. In this case, the diffusional and migrational flux oppose one another, and the net flux is governed by the dominant term in Equation (1). Consequently, staircase-like current decreases were not observed.

The step current magnitude associated with *Chlorella* collisions at the electrode surface was simulated using COMSOL Multiphysics to estimate the size of the colliding *Chlorella* cells. The simulation was performed in two-dimensional axisymmetric mode, with a spherical particle positioned at the center of an electrode of 5 µm radius immersed in electrolyte solution. The bulk concentration was set to 1 mM to match the experimental FcMeOH concentration, and the diffusion coefficient was set to 7.6 × 10^−10^ m^2^·s^−1^ based on the transport properties of FcMeOH [46,47]. Under these boundary conditions, the steady-state current at the electrode was calculated as a function of the particle size. The magnitude of the current decrease caused by particle collisions was determined by subtracting the steady-state current calculated in the presence of a particle from that obtained in the absence of a particle. These simulated current decreases were plotted as a function of particle size to generate a calibration curve, and the size of the colliding *Chlorella* cells was subsequently calculated by substituting the experimentally measured step currents into this calibration curve. The equation for the calibration curve is expressed as follows:

(2) $$y=\left(1.1\times 10^{-11}\right)e^{0.45x}-\left(1.81\times 10^{-11}\right)x\geq 1.0\;\mu m$$

**Figure 4:**
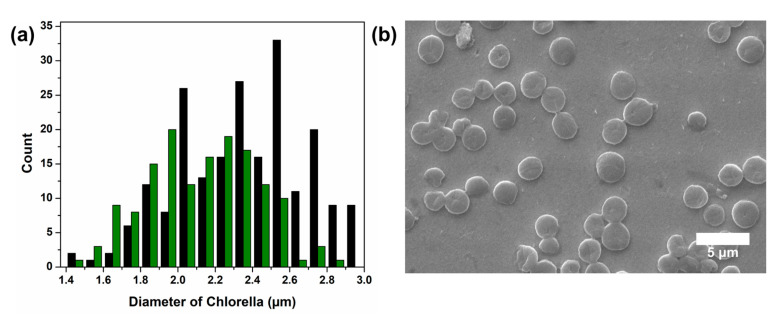
(**a**) Comparison of *Chlorella* size distributions obtained from electrochemical collision experiments (black bar) and SEM analysis (green bar). The electrochemical sizes were estimated by fitting the experimental step currents measured in FcMeOH solution to a calibration curve derived from COMSOL Multiphysics simulations. (**b**) SEM image of *Chlorella* cells.

Figure 4a compares the physical *Chlorella* size distribution measured by SEM with the electrochemical size distribution obtained by applying the simulation-based calibration curve to the step currents observed in the FcMeOH solution. The SEM-based size distribution was obtained by measuring the diameters of 150 randomly selected *Chlorella* cells. Comparison of the two distributions gives an average diameter of 2.34 µm from the simulation-based method and 2.11 µm from the SEM measurements, indicating that the electrochemical estimates are slightly larger than the physical measurements. This overestimation arises because the simulation assumes collision at the center of the electrode, whereas experimental collisions occur randomly across the surface, including near the edge. When a particle collides near the edge of a UME, mass transfer is hindered more strongly because of the radial diffusion profile (the so-called edge effect), resulting in a larger current decrease than for a central collision of a particle of the same size. Consequently, using these amplified current decreases to estimate size leads to values that exceed the actual dimensions. In addition, given that *Chlorella* cells have a negative surface charge and the redox probe ([Fe(CN)_6_]^4−^) is anionic, the overlap of electric double layers within the interstitial spaces between adjacent cells attached to the electrode creates an electrostatic barrier. This repulsion effect hinders the diffusion of redox probe molecules toward the electrode surface, potentially leading to a larger current decrease than that attributed solely to the geometric collision area. Considering these effects, the ~200 nm difference between the mean values indicates that the simulation-based method remains a viable approach for estimating *Chlorella* size. Figure 4b presents an SEM image of the *Chlorella* cells, revealing a flattened circular morphology. The cells may appear somewhat flattened relative to their hydrated state because the samples were dried for more than 24 h prior to imaging. The drying process may also contribute to underestimation of *Chlorella* size in the SEM measurement.

**Figure 5:**
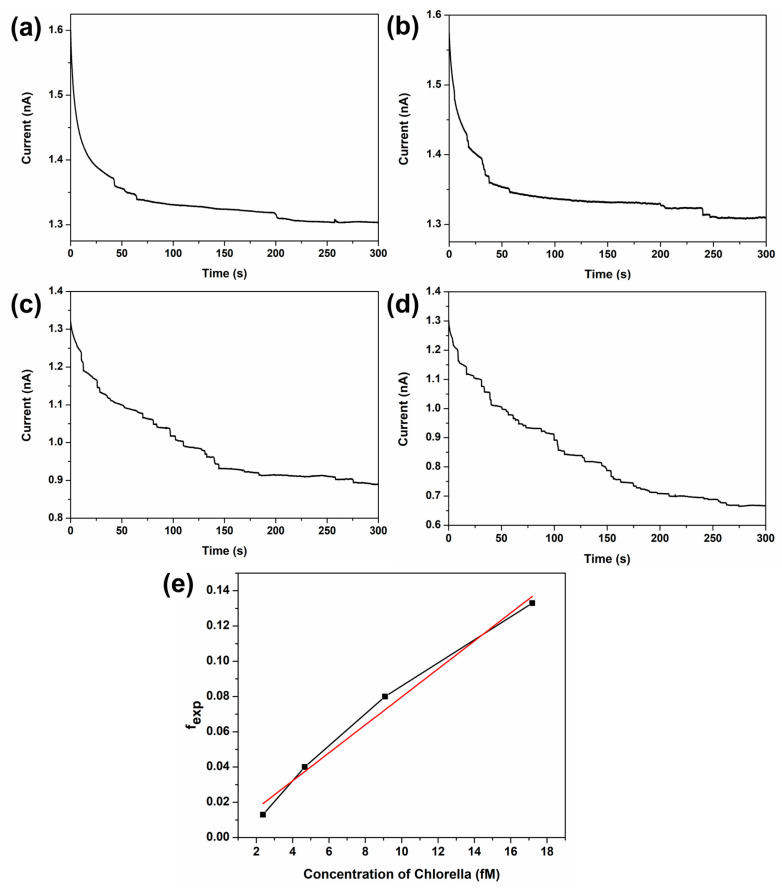
Amperometric *I*–*t* curves were obtained at +0.5 V (vs. Ag/AgCl) using a 10 µm Pt-UME in aqueous solution containing 1 mM FcMeOH and 0.5 mM KNO_3_ with *Chlorella* of (**a**) 2.37 fM, (**b**) 4.67 fM, (**c**) 9.08 fM, and (**d**) 17.2 fM. (**e**) Plot of the $f_{exp}$ with respect to the *Chlorella* concentration (black line) and linear calibration curve (red line).

Estimating the physical dimensions of *Chlorella* cells via simulation enables assessment of their growth stage in aquatic environments. Furthermore, quantifying cell population allows monitoring of algal bloom progression. Figure 5 presents collision experiments performed at various *Chlorella* concentrations in the FcMeOH solution. The *Chlorella* concentration was determined from UV-Vis absorbance measurements using the Beer–Lambert law (A=εlc). For the calculation, the absorbance (A) of *Chlorella* at 685 nm was used. The molar absorption coefficient (ε) was taken as 8.016 × 10^13^ L·mol^−1^·cm^−1^, and the optical path length (l) was 1 cm. The frequency of step currents attributed to *Chlorella* collisions increased with *Chlorella* concentration. In the diffusion-controlled regime, the collision frequency of single entities is proportional to their concentration, as described by the following equation [48].

(3) $$f_{diff}=4DCr_{UME}N_{A}$$

In this equation, D is the diffusion coefficient of the single entity, C is the concentration of the single entity, $r_{UME}$ is the electrode radius, and $N_{A}$ is the Avogadro’s constant. This model assumes diffusion-controlled transport, although charged particles are also transported by migration. To evaluate the origin of collisional flux quantitatively, the diffusion-controlled collision frequency was calculated by substituting the *Chlorella* concentration and diffusion coefficient into the equation. The diffusion coefficient of *Chlorella* was estimated using the Stokes–Einstein equation, assuming a spherical particle of 2 µm diameter. The Stokes–Einstein equation is as follows:

(4) $$D=\frac{k_{B}T}{6\pi \eta r}$$

*k_B_* represents the Boltzmann constant, T is the absolute temperature, η denotes the viscosity of water, and r corresponds to the radius of *Chlorella*. The calculated diffusion-controlled collision frequency ($f_{diff}$) is presented in Table 1. The theoretical diffusion-controlled collision frequency ($f_{diff}$) was approximately 2000 to 3000 times smaller than the experimentally observed frequency ($f_{exp}$). This substantial deviation indicates that diffusion alone cannot account for the observed collision frequency of *Chlorella* at the electrode.

Collision frequency arising from migration can be estimated quantitatively by calculating the transference number of the charged single entity [49]. This method relies on the premise that the total current at the electrode equals the sum of the partial currents associated with transport of all ions and charged single entities. In the equation, $z_{j}$ represents the effective charge of the specific species j, $u_{j}$ denotes its mobility, $C_{j}$ is the concentration of the species j, $I_{avg}$ is the average current of experiment, $N_{A}$ is the Avogadro’s constant, and F is the Faraday constant.

(5) $$t_{j}=\frac{\left|z_{j}\right|u_{j}C_{j}}{\sum_{i}\left|z_{i}\right|u_{i}C_{i}}$$

(6) $$f_{mig}=\frac{t_{cell}I_{avg}N_{A}}{z_{cell}F}$$

In Equation (5), the transference number ($t_{j}$) represents the fraction of the total current carried by each ionic species in the electrolyte. The transference number of *Chlorella* ($t_{cell}$), although negligible in the overall charge balance, corresponds to its relative flux toward the electrode in the electrolyte solution. The calculation of $t_{j}$ considers all species in the electrolyte solution, K^+^, NO_3_^−^, and *Chlorella*. FcMeOH was excluded from this calculation because it is electrically neutral in near-neutral pH condition. The migration-driven collision frequency ($f_{mig}$) was estimated by determining the partial current associated with *Chlorella* transport, which is the product of its transference number ($t_{cell}$) by the average total current ($I_{avg}$). This partial current was then divided by the effective charge of the cell ($z_{cell}$) and the Faraday constant (*F*) and subsequently multiplied by Avogadro’s number (*N_A_*) to yield the frequency of collision events by migration. For this calculation, the mobility of *Chlorella* was estimated using the Einstein–Smoluchowski equation to be 9.55 × 10^−8^ m^2^·V^−1^·s^−1^, and the effective charge was assumed to be −10,000. The equation is as follows:

(7) $$u_{j}=\frac{\left|z_{j}\right|FD_{j}}{RT}$$

*z_j_* is the effective charge of the species j, $D_{j}$ represents its diffusion coefficient, F is the Faraday constant, R, and T are the gas constant, and the absolute temperature, respectively. The resulting $f_{mig}$ values are listed in Table 1. The calculated $f_{mig}$ values are comparable to the experimental values ($f_{exp}$), in contrast to the diffusion-controlled estimates ($f_{diff}$). Thus, *Chlorella* collisions at the electrode are governed primarily by migration. The theoretical migration-to-diffusion collision frequency ratio ($f_{mig}/f_{diff}$), which is approximately 3400 in this system, quantifies the magnitude of the migration effect and confirms its dominance over diffusion.

Finally, Figure 5e plots the $f_{exp}$ values as a function of *Chlorella* concentration. The red line in Figure 5e represents the calibration curve for this data, and its equation is expressed as follows:

(8) y=0.0079x+0.0005

In this equation, x represents the concentration in fM. The standard deviation (s) was obtained from repeated measurements at the lowest tested concentration (2.37 fM), giving s=0.00248. The limit of detection (LOD) was calculated using the standard criterion LOD=3 s/m, where m is the slope of the calibration curve, resulting in an LOD of 0.94 fM. This equation enables the reverse estimation of *Chlorella* concentration based on the collision frequency observed under the experimental conditions. Collectively, these findings demonstrate the proposed electrochemical collision method enables simultaneous determination of *Chlorella* cells concentration and size.

Collectively, these findings demonstrate that the proposed electrochemical collision method enables simultaneous determination of the concentration and size of *Chlorella* cells. It should be noted, however, that complete blocking of the UME surface is not expected under these conditions. Upon collision, *Chlorella* cells are likely to form a three-dimensional, randomly stacked layer on the electrode surface, within which interstitial voids remain. These void spaces permit continued diffusion of FcMeOH to the electrode, thereby preventing full suppression of the background current. This interpretation is consistent with the experimental observations, as the background current does not decrease below approximately 10% of its initial value, even at high collision frequencies (Figure 5). Such partial blocking behavior is therefore intrinsic to the cell geometry and stacking configuration rather than a limitation of the electrochemical detection method.

**Figure 6:**
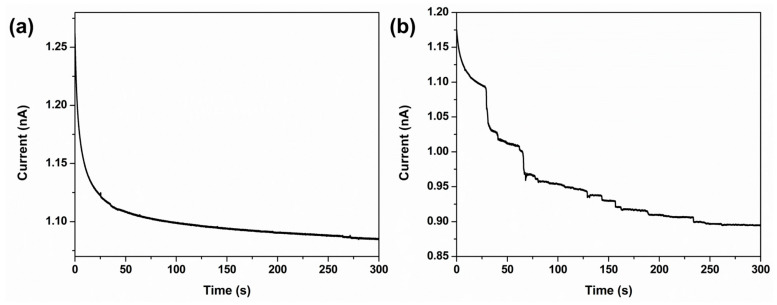
Amperometric *I*–*t* curves were obtained using a 10 µm Pt-UME in aqueous solution containing 1 mM FcMeOH and 0.5 mM KNO_3_ after the injection of (**a**) lake water without *Chlorella* and (**b**) lake water with *Chlorella* at +0.5 V (vs. Ag/AgCl).

To further evaluate the robustness of the proposed method in complex matrices, additional collision experiments were conducted using water samples collected from a local lake. Figure 6a shows the *I*–*t* curve recorded at an applied potential of 0.5 V after injecting lake water without *Chlorella* into an electrolyte containing 1 mM FcMeOH and 0.5 mM KNO_3_. Under these conditions, no staircase-like current decreases were observed, indicating that the lake water matrix itself does not produce false-positive collision signals. In contrast, when *Chlorella* cells were introduced into the same lake water sample and injected into the electrolyte (Figure 6b), distinct staircase current decreases corresponding to collision events were clearly detected. These results demonstrate that the proposed electrochemical collision strategy remains effective in real environmental samples containing coexisting ions and potential contaminants, confirming its applicability in complex matrices.

## 4. Conclusions

In this study, the electrochemical detection of living *Chlorella* cells was demonstrated using the single-particle collision method at a UME. The principle of this method relies on blockage of the redox probe flux toward the UME by individual *Chlorella* cells, resulting in discrete staircase-like current decreases. The role of migration was evaluated in terms of cell collision flux and retention at the electrode surface, which increases sensitivity for negatively charged microalgae. To optimize the detection system, multiple redox probes were examined. Oxidation of electrically neutral FcMeOH oxidation generated stronger electrostatic attraction than oxidation of ionic ferrocyanide because of differences in ionic strength, thereby enhancing cell transport to the electrode surface. Diffusion-controlled experiments, conducted by increasing the supporting electrolyte concentration, showed that diffusion alone does not produce a staircase current response in this system because the cells are not effectively retained at the electrode surface. Furthermore, the migration-driven collision frequency calculated from the transference number and mobility of *Chlorella* agreed with the experimental values. The migration-to-diffusion collision ratio provides a quantitative measure of migrational strength and can guide the design of migration-controlled detection system. Using the calibration curve, *Chlorella* concentration can be determined from the measured collision frequency. In addition, COMSOL Multiphysics simulations were employed to correlate the magnitude of current steps with cell size. By simultaneously determining both the size and concentration of algal cells, this methodology may provide an early warning platform for monitoring the progression of HABs in aquatic ecosystems.

## Figures and Tables

**Table 1:** Collision frequency according to *Chlorella* concentration.

*Chlorella* Concentration (fM)	f_diff_ (s^−1^)	f_mig_ (s^−1^)	f_exp_ (s^−1^)
2.37	7.00 × 10^−6^	0.024	0.013
4.67	1.38 × 10^−5^	0.047	0.040
9.08	2.68 × 10^−5^	0.092	0.080
17.2	5.08 × 10^−5^	0.175	0.133

## Data Availability

Data are contained within the article.

## Abbreviations

The following abbreviations are used in this manuscript:

CV	Cyclic voltammogram
UME	Ultramicroelectrode
FcMeOH	Ferrocene methanol
HAB	Harmful Algal Bloom
